# Supplementary material for: Metabolic rate evolves rapidly and in parallel with the pace of life history
Source: Nat Commun. 2018 Jan 2;9:14. doi: 10.1038/s41467-017-02514-z (PMC5750215; doi:10.1038/s41467-017-02514-z)
Supplement: Supplementary file 1 — Supplementary Information [file 41467_2017_2514_MOESM1_ESM.pdf]

Supplementary Table 1. Means (±1SE) and sample sizes (n) for standard metabolic rate (SMR) and body mass in six populations of Trinidadian guppies (*Poecilia reticulata*) from three different drainages in Trinidad, West Indies that were reared as F1 offspring under common garden conditions in the laboratory. Values for standard metabolic rate are standardized to a common body mass of 74 mg (mean across all fish). The range (minimum to maximum) is also given for body mass. Included are populations from low (LP) and high (HP) predation sites that have evolved a slow (Quare and Yarra-Limon) versus fast-paced (Oropuche and Yarra) life history in the Yarra and Oropuche drainages as well as a naturally-occurring population with a fast-paced life history (El Cedro) and its descendants (El Cedro Intro) that evolved a slow-paced life history since being transplanted to an upstream low predation site 35 years ago in the Caroni drainage.

	SMR (mg O2 h^{-1})	Body mass (mg)		
Locality	Mean (±1SE)	Mean (±1SE)	Range	n
Oropuche drainage				
Quare LP	0.017 (0.001)	83.5 (3.9)	43-116	29
Oropuche HP	0.025 (0.001)	75.7 (3.2)	41-116	28
Yarra drainage				
Yarra-Limon LP	0.023 (0.001)	81.5 (4.6)	45-125	26
Yarra HP	0.028 (0.001)	64.5 (3.8)	32-106	28
Caroni drainage				
El Cedro Intro LP	0.019 (0.001)	63.6 (3.9)	38-119	22
El Cedro HP	0.026 (0.001)	76.5 (5.0)	37-134	24

Supplementary Table 2. Life history traits of Trinidadian guppy (*Poecilia reticulata*) populations. Included are populations from low (LP) and high (HP) predation sites that have evolved a slow (Quare and Yarra-Limon) versus fast-paced (Oropuche and Yarra) life history in the Yarra and Oropuche drainages as well as a naturally-occurring population with a fast-paced life history (El Cedro) and its descendants (El Cedro Intro) that evolved a slow-paced life history since being transplanted to an upstream low predation site 35 years ago in the Caroni drainage. Data are from previous common garden laboratory studies of F2 generation fish[1,2].

Locality	Male age at maturity (days)	Male size at maturity (mg)	Female age at first parturition (days)	Female size at first parturition (mg)	Inter-litter interval (days)	RA* (%)
Oropuche drainage						
Quare LP	60.10	97.60	89.20	296.70	24.70	11.72
Oropuche HP	49.70	83.20	75.00	209.00	21.60	13.45
Yarra drainage						
Yarra-Limon LP	58.00	73.92	90.50	152.92	24.77	14.90
Yarra HP	45.12	55.06	73.20	131.60	22.03	20.06
Caroni drainage						
El Cedro Intro LP	69.17	75.70	88.85	173.20	25.60	16.16
El Cedro HP	60.19	68.65	82.70	166.13	25.25	17.64

* RA = reproductive allotment (% dry weight devoted to offspring)

Supplementary Table 3. Linear mixed models testing the effects of female reproductive status (no eggs versus eggs or embryos) on standard metabolic rate (mg O_2 h^{-1}) of Trinidadian guppy (*Poecilia reticulata*) populations with a slow versus fast-paced life history from the Yarra, Oropuche and Caroni drainages. Both standard metabolic rate and body mass were log_{10}-transformed. See main text for detailed description of models.

	Tests of fixed effects		
Predictors	df	*F*	*P*
Body mass	1,55.0	40.19	<0.001
Reproductive status	1,70.0	0.11	0.737
Life history	2,67.6	10.34	<0.001
Drainage	2,63.0	10.04	<0.001
Reproductive status x Life history	2,65.9	0.56	0.574
Reproductive status x Drainage	2,65.9	0.01	0.996
Drainage x Life history	1,60.0	1.74	0.193

Supplementary Note 1: Life history evolution in Trinidadian guppies

Our research on the evolution of life histories in guppies was inspired by the earlier discovery of high versus low predation communities in Trinidadian streams. Haskins et al.[3] and Endler[4] described the gradient from downstream high predation to upstream low predation , plus showed the same progression of fish communities was found in multiple rivers draining the south slope of the Northern Range Mountains. They also discovered the barrier waterfalls that often served as boundaries to the upstream dispersal of some fish species, creating high versus low predation communities found in close proximity to one another. Both authors also discovered and characterized the differences in male coloration in high versus low predation communities. Male guppies tend to be more brightly coloured in low predation communities. Endler argued that this pattern was the product of selection by predators, who were better able to target and capture brightly coloured males, versus females who were more attracted to and more likely to mate with brightly coloured males. Female preferences dominate in the absence of predators, causing the males to evolve brighter colour patterns. Our insight was to realize that these patterns could only evolve if there were higher adult mortality rates in localities with predators. If such differences in mortality risk were present, then they also provided a link to the theory of life history evolution, which modelled how life histories should evolve in response to higher or lower mortality risk. The prediction, well-articulated even in the 1970's, was that high mortality rates should select for "faster" life histories, meaning earlier reproduction and a higher rate of investment in reproduction, as opposed to growth or maintenance[5-7]. By 1982, Endler also showed that it was possible to execute experimental studies of evolution on natural populations. He introduced guppies over a barrier waterfall that had excluded guppies and predators. In this way, he created a descendant population occupying a low predation environment derived from high predation ancestors. He performed the introduction in 1976 and found that these males had evolved brighter colour patterns than their ancestors (the control) by 1980[8].

We first sampled guppies from high and low predation environments distributed across several streams draining the south slope of the Northern Range Mountains[9]. Dissections of wild-caught fish revealed that those from high predation environments began to reproduce at a smaller size, produced more offspring per litter and invested more resources in each litter than did their counterparts from low predation localities. All of these differences are consistent with a "faster" life history, such that the guppies adapted to life with predators matured at an earlier age and invested more in reproduction. We then reared representatives from two high and two low predation communities in the laboratory for two generations and quantified the life histories of the grandchildren of wild-caught adults in a setting that controlled for the physical environment and the quantity of food consumed by each individual[1]. Here we showed that male and female guppies from high predation environments are younger and smaller at sexual maturity. Female guppies from high predation environments also reproduce more often, with the interval between the birth of each successive litter of liveborn young being 10% shorter than that of guppies from low predation environments. High predation females also produce more offspring per litter and the individual offspring are smaller than those produced by females adapted to low predation environments. Female guppies adapted to life with predators thus invest more in reproduction because of differences in timing and in the quantity of resources devoted to each litter. In all regards, the results therefore show that guppies adapted to high predation environments have faster life histories than those adapted to low predation environments.

We later performed experiments in which we hybridized guppies from high versus low predation environments to confirm the genetic basis of offspring size differences[10]. We also developed methods for estimating mortality risk in natural populations and showed that

guppies from high predation environments do indeed experience much higher mortality rates than their counterparts from low predation environments[11].

We then extended our contrast between high and low predation environments to the north slope of the Northern Range Mountains, which are inhabited by a different fish fauna, yet retain the contrast between high versus low predation environments. Rivers on the south slope were formerly tributaries to rivers in South America and have a faunal composition typical of the mainland, dominated by cichlids and characins. Rivers of the north slope never had a mainland connection and are instead dominated by fish derived from marine environments, including gobies and mullets. We found that guppies from high and low predation environments on the north slope have the same fast-slow differences in life histories as those from the south slope. We characterized these differences, and the parallel nature of the north and south slopes, in the same fashion as in our original work on the south slope. We first compared the life history phenotypes of wild-caught guppies from many high versus low predation environments on the north slope[12], then characterized the life histories of the grandchildren of representatives from high versus low predation environments from two rivers in a laboratory setting[2]. The north slope fish from high predation environments had faster life histories than those from low predation environments in all of the same ways as seen on the south slope: they were younger and smaller at maturity, females had shorter time intervals between successive broods of young, and they produced more babies per litter, invested more resources in each litter, and produced smaller offspring. The fact that these differences in life histories were so well replicated in the face of a different fauna of predators, but one that shared the differences in risk of predation, makes a stronger case for mortality risk as the causal agent of life history evolution. More importantly, it shows that there is a strong and consistent relationship between adaptation to life with predators and having a faster life history.

We also initiated experimental studies of life history evolution that were patterned after Endler's 1976 experiment. In this context, we showed that faster life histories will evolve as predicted in response to manipulations of mortality risk in natural populations. In one type of experiment, we transplanted guppies from a high predation environment below a barrier waterfall into a previously guppy-free low predation environment above a barrier. In a second type of experiment we transplanted predators from below a barrier waterfall that excluded predators but not guppies. The introduced predators were confined to a short stretch of stream by additional waterfalls. In all cases, the guppy life histories evolved in a fashion that was consistent with the comparisons of fish from natural high and low predation environments. Our inference of evolution was derived from laboratory experiments performed on the grandchildren of wild caught fish from the experimental and control populations. Guppies transplanted from high to low predation environments evolved delayed ages and larger sizes at maturity. Females also evolved to produce fewer, larger offspring and to invest less in their first litters of young[13-15]. In addition, guppies exposed to introduced predators evolved earlier ages and smaller sizes at maturity[16].

Together, these experimental studies reinforce our argument that life history traits evolve as a tightly interconnected suite of traits and that the fast-slow life histories of guppies from high versus low predation environments is an adaptation to differences among environments in the risk of predation.

Supplementary References

1 Reznick, D. N. The impact of predation on life history evolution in Trinidadian guppies: Genetic basis of observed life history patterns. *Evolution* **36**, 1236-1250 (1982).

2 Reznick, D. N. & Bryga, H. A. Life-history evolution in guppies (Poecilia reticulata: Poeciliidae). 5. Genetic basis of parallelism in life histories. *The American Naturalist* **147**, 339-359 (1996).

3 Haskins, C. P., Haskins, E. G., McLaughlin, J. J. A. & Hewitt, R. E. in *Vertebrate Speciation* (ed W. F. Blair) (University of Texas Press, 1961).

4 Endler, J. A. A predator's view of animal color patterns. *Evolutionary Biology* **11**, 319-364 (1978).

5 Gadgil, M. & Bossert, P. W. Life historical consequences of natural selection. *American Naturalist* **104**, 1-24 (1970).

6 Law, R. Optimal life histories under age-specific predation. *American Naturalist* **114**, 399-417 (1979).

7 Michod, R. E. Evolution of life histories in response to age-specific mortality factors. *American Naturalist* **113**, 531-550 (1979).

8 Endler, J. A. Natural selection on color patterns in Poecilia reticulata. *Evolution* **34**, 76-91 (1980).

9 Reznick, D. N. & Endler, J. A. The impact of predation on life history evolution in Trinidadian guppies (*Poecilia reticulata*). *Evolution* **36**, 160-177 (1982).

10 Reznick, D. Genetic determination of offspring size in the guppy (Poecilia reticulata). *American Naturalist* **120**, 181-188 (1982).

11 Reznick, D. N., Butler M. J., I., Rodd, F. H. & Ross, P. Life history evolution in guppies (Poecilia reticulata). 6. Differential mortality as a mechanism for natural selection. *Evolution* **50**, 1651-1660 (1996).

12 Reznick, D. N., Rodd, F. H. & Cardenas, M. Life-history evolution in guppies (Poecilia reticulata: Poeciliidae). IV. Parallelism in life-history phenotypes. *American Naturalist* **147**, 319-338 (1996).

13 Reznick, D. N. & Bryga, H. Life-history evolution in guppies. 1. Phenotypic and genotypic changes in an introduction experiment. *Evolution* **41**, 1370-1385 (1987).

14 Reznick, D. A., Bryga, H. & Endler, J. A. Experimentally induced life-history evolution in a natural population. *Nature* **346**, 357-359 (1990).

15 Reznick, D. N., Shaw, F. H., Rodd, F. H. & Shaw, R. G. Evaluation of the rate of evolution in natural populations of guppies (Poecilia reticulata). *Science* **275**, 1934-1937 (1997).

16 Reznick, D. N., Buckwalter, G., Groff, J. & Elder, D. The evolution of senescence in natural populations of guppies (Poecilia reticulata): a comparative approach. *Experimental Gerontology* **36**, 791-812 (2001).
